# Conventional vs. Organically Produced Honey—Are There Differences in Physicochemical, Nutritional and Sensory Characteristics?

**DOI:** 10.3390/foods13223573

**Published:** 2024-11-08

**Authors:** Sladjana P. Stanojević, Danijel D. Milinčić, Nataša Smiljanić, Mirjana B. Pešić, Nebojša M. Nedić, Stefan Kolašinac, Biljana Dojčinović, Zora Dajić-Stevanović, Aleksandar Ž. Kostić

**Affiliations:** 1 Department of Food Technology and Biochemistry, Faculty of Agriculture, University of Belgrade, Nemanjina 6, 11080 Belgrade, Serbia; danijel.milincic@agrif.bg.ac.rs (D.D.M.); akismiljanic@gmail.com (N.S.); mpesic@agrif.bg.ac.rs (M.B.P.); akostic@agrif.bg.ac.rs (A.Ž.K.); 2 Department of Breeding and Reproduction of Domestic and Farmed Animals, Faculty of Agriculture, University of Belgrade, Nemanjina 6, 11080 Belgrade, Serbia; nedicn@agrif.bg.ac.rs; 3 Department of Agrobotany, Faculty of Agriculture, University of Belgrade, Nemanjina 6, 11080 Belgrade, Serbia; stefan.kolasinac@agrif.bg.ac.rs (S.K.); zoradajic@agrif.bg.ac.rs (Z.D.-S.); 4 Institute of Chemistry, Technology and Metallurgy National Institute of the Republic of Serbia, University of Belgrade, Njegoševa 12, 11000 Belgrade, Serbia; bmatic@chem.bg.ac.rs

**Keywords:** linden/acacia/chestnut/meadow honey, production method, physicochemical properties, mineral composition, phenolic components, antioxidant potential, Raman spectroscopy, sensory characteristics

## Abstract

Honey is a sweet syrup mixture substance produced by honey bees. Contradictory results have been reported on the influence of organic and conventional beekeeping on the properties of honey. The aim of this research was to determine the potential difference between organically and conventionally produced honey of the same botanical origin (linden, acacia, chestnut, meadow). It was shown that the electrical conductivity (0.16–0.98 mS/cm), optical rotation (−1.00 − (−2.60) [α]_D_^20^), pH values (3.30–4.95), free acidity (4.0–9.0 mmol/kg), total content of phenolic (76.5–145.9 μg GAE/g dry weight (d.w.)) and flavonoids (48.7–307.0 μg QE/g d.w.), antioxidant potential, phenolic profile, mineral composition, color (−8.62–126.57 mmPfund) and sensory characteristics, although statistically significant differences were found, were not significantly improved better in the organic samples. All organic honey samples were richer in hydroxycinnamic acid derivatives (60.5–112.1 μg CGAE/g d.w.) compared to conventional honey (56.7–91.1 μg CGAE/g d.w.) of the corresponding botanical origin. The results show that organic beekeeping does not lead to the production of honey with significantly better physicochemical, nutritional and sensory properties compared to conventionally produced honey.

## 1. Introduction

“Honey is the natural sweet substance produced by honey bees from the nectar of plants or from secretions of living parts of plants or excretions of plant-sucking insects on the living parts of plants, which the bees collect, transform by combining with specific substances of their own, deposit, dehydrate, store and leave in the honeycomb to ripen and mature” [1]. Honey is a food product that has been used in human nutrition since ancient times. The earliest evidence of beekeeping by primitive man is painted on the cave walls in Spain, Africa and India, 8000 before the new era. The ancient civilizations of the Egyptians, Greeks and Romans have left traces of the use of honey on monuments, in pyramids and in the works of Greek philosophers and writers [2]. Honey is used not only as a nutritional product but also in traditional medicine and clinical conditions. It has been found that the constituents of honey may have antioxidant, antiproliferative, antimicrobial, anticancer, antimetastatic and antiinflammatory properties [3]. However, if honey is contaminated, it can pose serious risks to human health [4]. In an effort to preserve the quality of honey produced in a traditional way without the use of chemicals in an unpolluted environment, there has been an increase in organic honey production.

Due to the protection of the environment, biodiversity, and human health, the production of organic food is experiencing great growth around the world. Organic honey production requires that the honey-bearing pastures are not exposed to chemicals, pesticides are present in the air/soil or antibiotics, and the bees are not fed with sugar, which is common in conventional beekeeping. Organic beekeeping uses beehives made of natural material that do not pose a risk to the environment or bee products and do not harm the bees. The organic apiary must be located within a radius of 3 km from roads and conventionally cultivated crops. The conversion from a conventional to an organic apiary takes one year [5].

Conventional beekeeping allows the use of a wide range of pesticides for which maximum concentrations in honey are set [6]. In addition, they are prescribed by the values for the maximum allowed concentrations for residues of pharmacologically active substances in honey [7]. Inadequate use of these active substances makes honey a threat to human health.

It is known that the variability of the chemical composition of honey is dependent on many factors, including botanical origin (variety), geographical (place of production) and apiary conditions (including the production system). There are many studies on the differences between honey based on botanical and geographical origin, as well as on agro-technical measures applied in beekeeping and the influence of honey storage conditions or the environment. In contrast, very limited research has been reported on the influence of organic and conventional beekeeping on the characteristics of honey, with conflicting results [8,9,10]. In addition to the fact that organically produced honey must not contain pesticides and various pharmacological preparations, it is generally assumed that organically-produced honey has a higher nutritional value. But is it really the case? The aim of this research was to determine the potential difference in the physical-chemical, nutritional and sensory characteristics between honey of the same botanical origin produced in organic and conventional beekeeping.

## 2. Material and Methods

### 2.1. Sample Collection

Eight samples of honey produced using organic and conventional beekeeping by certified beekeepers were used for the study, differing in their botanical origin (linden, acacia, chestnut, meadow) and their geographical origin from the Balkans (Table S1). These 4 organic and conventional samples have been chosen because they are the most represented on our market. All analytical methods were performed within a maximum of three months, during which time the samples were stored in a freezer, while the sensory analysis was conducted immediately after sample collection. Their freshness at the time of analysis was verified through the determination of hydroxymethylfurfural (<20 mg/kg). The pollen frequency in honey samples was not examined. Plant source was declared by beekeepers who tested honey samples in the certified laboratory (for chestnut honey, 79% and other tested samples >60% of a specific pollen type). All tested samples of organic honey had a certificate from a licensed laboratory that they belonged to the “organic honey” group.

### 2.2. Analytical Methods

The color and optical density of honey were determined by spectrophotometric measurement of the absorbance (635 nm) of an aqueous honey solution (1:1; *w*/*v*) using the Pfund scale after conversion of absorbance values [11]. Other physicochemical parameters were analyzed using IHC methods [12]. The moisture content and soluble solids of the honey samples were determined at 20 °C using an Abbe-type refractometer (Digital Refractometer, Atago Co., Ltd., Tokyo, Japan) and expressed in °Brix. The free acidity of honey is the content of all free acids, determined by the titrimetric method (by titration to pH 8.3) and expressed in millimoles of acid/kg of honey (mmol/kg; [13]). Honey pH value (in 10% aqueous honey solution) and free acidity were determined using pH meter-Consort-C931 (Turnhout, Belgium). The specific optical rotation was measured with an Atago^TM^Polax-2L polarimeter (Tokyo, Japan) and expressed as the angle of rotation of polarized light at the wavelength of the sodium-D line at 20 °C ([α]_D_^20^) of an aqueous honey solution of 1 dm depth containing 1 g/mL of the substance. Electrical conductivity was determined using a 20% (*w*/*v*) aqueous honey solution at 20 °C (Jenway Conductivity Meter 4310; Stone, UK) and expressed in milliSiemens per centimeter (mS/cm). The diastase activity of the samples was measured using the Phadebas method [12], and the results were expressed in diastase number (DN).

The total phenolic (TPC) and total flavonoids (TFC) content, as well as the content of hydroxycinnamon acid derivatives (DHCA), was determined using spectrophotometric methods (UV-1800, Shimadzu USA Manufacturing Inc, Canby, OR, SAD) according to Kostić et al. [14]. The results were expressed: for TPC as micrograms of gallic acid equivalents per gram of dry sample (μgGAE/g), for TFC as micrograms of quercetin per gram of dry sample (μgQE/g) and for DHCA as micrograms of chlorogenic acid equivalents per gram of dry sample (μgCGAE/g).

The profile of phenolic compounds was analyzed by UHPLC Q-ToF MS analysis in aqueous honey solution (1:2; *w*/*w*) on an Agilent 1290 Infinity ultra-high-performance liquid chromatography (UHPLC) system coupled with a quadrupole time-of-flight mass spectrometry (6530C Q-ToF-MS) from Agilent Technologies, Inc. Santa Clara, CA, USA, using the same method and operating parameters as previously described in detail by Kostić et al. [14]. Data-dependent acquisition (DDA) was employed for suspect screening, using the Auto MS/MS acquisition mode (100–1700 *m*/*z*; scan rate 1 spectra/sec), with fixed collision energy set at 30 eV. Agilent MassHunter software (https://www.agilent.com.cn/en/promotions/masshunter-mass-spec) was used for instrument control and data analysis. Phenolic compounds and abscisic acid were quantified using available standards and expressed as µg/g honey. Table S2 shows a list of phenolic compounds used for quantification and their equation parameters. The exact masses of the components were calculated using ChemDraw software (version 12.0, CambridgeSoft, Cambridge, MA, USA).

The antioxidant properties were determined using three methods: DPPH (α,α-diphenyl-2-picrylhydrazyl) radical scavenging activity, ferric-reducing power (FRP), cupric-reducing antioxidant capacity (CUPRAC) by the procedure detailed by Kostić et al. [14,15]. The results were expressed as milligram equivalents of ascorbic acid per gram of dry sample (mgAAE/g) for the FRP and CUPRAC assays and as a percentage of radical inhibition for the DPPH assay.

The concentrations of macro and microelements in the solution obtained after total mineralization were measured using inductively coupled plasma with optical emission spectrometry (ICP-OES) on the Thermo Scientific iCAP 6500 Duo ICP instrument (Thermo Fisher Scientific, Cambridge, UK) with iTEVA software (https://iteva.software.informer.com/). Calibration standard solutions were prepared from three certified standards: Multi-Element Plasma Standard Solution 4, Specpure^®^ (Alfa Aesar GmbH & Co. KG, Emmerich am Rhein, Germany); SS-Low Level Elements ICV Stock and ILM 05.2 ICS Stock 1 (VHG Labs, Inc-Part of LGC Standards, Manchester, NH, USA). Quantification was performed in triplicate on emission lines with minimal spectral interference. The relative standard deviation was RSD < 3%, with calibration curve correlation coefficients > 0.99. The limits of detection were LOD = 0.01–0.5 µg/L, and the limits of quantification were LOQ = 0.1–1 µg/L. Quality control (QC) included using two certified reference materials (CRMs): DORM 4 (NRCC, North Bay, ON, Canada) and EPA Method 200.7 LPC Solution (ULTRA Scientific, Manchester, NH, USA), with recovery of measured concentrations ranging from 98% to 103% [16].

Raman spectroscopy analysis was performed on the confocal Raman microspectroscopy Witec Alpha 300R (Dreieich, Germany) using a 785 nm laser with a power of 80 mW, an integration time of 60 s, and an objective with 10× magnification with a resolution of 1.24 cm^−1^ and the total magnification of 10×. Chemometric analysis of spectra was performed by each honey sample was recorded 36 times, and the final matrix was 288 × 1562 (number of spectra x number of variables). The spectral range between 300 cm^−1^ and 1500 cm^−1^ was selected for further analysis since being informative. The baseline correction, spectra normalization, and 2nd order derivative processing were applied to achieve the best discrimination power. After preprocessing, PCA was performed to reduce the number of variables. Quadratic Discriminant Analysis (QDA) was applied to develop a valid model for the classification of all tested samples, i.e., to obtain the separate classification groups corresponding with the total sample number (8 groups in total, i.e., Linden, Acacia, Chestnut and Meadow from organic and conventional production) and to be agreeable with their botanical origin (4 groups in total i.e., Linden, Acacia, Chestnut and Meadow from both production ways taken jointly). For this purpose, the 5 Principal components (PCs) were used. Validation of the model was performed using the Leave-One-Out-Cross-Validation (LOOCV) method. The chemometric analysis was performed using Unscrambler X software (version 10.4).

### 2.3. Sensory Analysis

The sensory evaluation was carried out by 10 trained expert panelists in two sessions according to the descriptive semi-quantitative method of. Marcazzan et al. [17]. For the evaluation of the overall acceptability, a “hedonic scale” was used with sixty honey consumers in two sessions, with acceptability grades ranging from 1 to 9 [18]. Sensory analyses were conducted in accordance with the Code of Professional Ethics of the University of Belgrade [19]. At the beginning of the sensory examination, all panelists gave written consent to participate, and they were aware they could withdraw from the study at any time, that their responses were confidential, that the responses would be used for scientific purposes, as well as that the participant’s data and their answers will not be published without their knowledge. Before sensory evaluation, participants were fully informed about study requirements. The tested samples were safe for consumption.

### 2.4. Statistical Analysis

The results of the study were expressed as the mean and pooled standard deviation (Pooled std) of three replicates (unless otherwise indicated). For that, Statistica software version 8.0 (StatSoft Co., Tulsa, OK, USA) was used, as well as for the determination of Pearson correlation coefficients and Tukey’s test (at *p* < 0.05). Principal component analysis (PCA) was used to determine the possible correlations between the measured objects.

## 3. Results and Discussion

### 3.1. Physicochemical Parameters

Physical parameters (such as electrical conductivity and specific optical rotation) are the basic characteristics of honey that are important for its classification; their measurement is comparatively simple, and they provide important information [20]. Optical rotation is a parameter that shows the botanical origin of the honey and indicates adulteration of the honey [21]. The examined samples of honey of different botanical origins (linden, acacia, chestnut, meadow) belong to the group of nectar-honey, which originate from the nectaries of flowers [22,23]. The values of optical rotation depend on the type of sugar and relative proportions of sugars in the honey. The specific rotation of fructose is −92.4°, glucose +52.7°, sucrose +66.5°, maltose +130.4°, melezitose +88.2° and erlose +121.8° [24,25]. Since nectar honey is dominated by fructose, which has a negative specific rotation, the total specific rotation of this type of honey is negative [24,25,26]. Accordingly, all honey samples examined in this study, as they belong to the nectar-honey group, should have negative values for specific optical rotation, which was also registered (for honey obtained by organic beekeeping—from −0.10 [α]_D_^20^ to −1.83 [α]_D_^20^ respectively; for honey obtained by conventional beekeeping—from −1.00 [α]_D_^20^ to −2.60 [α]_D_^20^ respectively; Table 1).

In addition to optical rotation, the electrical conductivity of honey is a parameter used to test the quality and botanical origin of honey [27]. A strong correlation was registered between these values (*r* = 0.81; Table S3). The greater the content of inorganic elements, organic acids, free amino acids, proteins and complex compounds in honey, the greater the electrical conductivity [28]. Since the mineral elements primarily enter the honey with the pollen, their content depends on the predominant pollen in the honey, which indicates its botanical origin [24]. The electrical conductivity values in the examined samples ranged from 0.16 to 0.98 mS/cm. The obtained results are in agreement with the literature data in which values for the electrical conductivity of honey from different origins of 0.15–1.64 mS/cm were recorded [29]. Organic meadow honey had the highest electrical conductivity value (0.98 mS/cm), while organic acacia honey had the lowest electrical conductivity value (0.16 mS/cm; Table 1). With the exception of acacia honey, all other tested samples of organic honey had higher electrical conductivity values than conventionally produced honey of the same origin. (Table 1). Electrical conductivity is defined by a new international standard for honey [1,22,30], replacing data for ash content in honey. According to the standard regulation [1], the electrical conductivity of honeydew and chestnut honey is above 0.8 mS/cm, while the electrical conductivity in nectar-honey is below 0.8 mS/cm. In the tested samples, the values for the electrical conductivity of organically produced meadow honey deviate from the prescribed value for flower honey. Namely, the value was higher than 0.8 (0.98 mS/cm Table 1). This indicates the possibility that the bees collected flower nectar and honeydew [23]. In the literature, values higher than 0.8 mS/cm are given for the electrical conductivity of meadow honey. For example, Živkov-Baloš and co-workers [31] examined eighteen samples of meadow honey from the Vojvodina region (Serbia) and registered an electrical conductivity in the range of 0.08–1.19 mS/cm. The obtained values for electrical conductivity and optical rotation were in the middle range depending on the moisture content of the examined samples (*r* = −0.54 and *r* = −0.57, respectively; Table S3).

Moisture content is the only compositional criterion of honey, which has to be fulfilled in the world honey trade as part of the Honey Standard [26]. Different moisture content was registered between honey samples of the same botanical origin and different methods of production (organic and conventional). In most of the tested honey (except for linden honey), a higher moisture content was registered in conventionally produced samples. The moisture content was determined to be 14.43–18.39% (Table 1) in the tested samples. The permitted moisture content in honey is up to 20%, according to the Rulebook on the quality of honey and other bee products [32]. A maximum value of 21% for the moisture content is according to the regulation of Codex Alimentarius [33], and the same value has been proposed by the European Commission [34] for the new standard. A higher moisture content can lead to the fermentation of the honey and the formation of acetic acid [26,35]; both processes are undesirable. At the same time, lower moisture content can lead to the development of caramelization and Maillard reactions during honey storage [35]. The values for the moisture content in all the tested samples were below the maximum value recommended in these regulations and in agreement with the values reported in the literature for the moisture content of honey of different botanical and geographical origins. For example, Escuredo et al. [36] studied 187 honey samples and registered an average moisture content of 16.9–18.0%. For example, Machado et al. [37] found a moisture content in the range of 14.2–18.0% when analyzing 51 honey samples. Some national beekeeping organizations (e.g., Belgium, Spain, Austria, Germany, Switzerland and Italy) prescribe maximum moisture content values of 17.5–18.5% for special classes of quality honey [26]. The moisture content of honey depends on the season and the degree of maturity of the honey that has been reached in the hive [38] as well as the botanical origin of the honey, the relative humidity in the room and the processing and storage conditions [24]. The moisture content can affect various parameters of honey quality, such as its crystallization quality, viscosity, solubility, taste, and color [36].

The moisture content correlated moderately with diastase enzyme activity in the tested samples (*r* = 0.63; Table S3). Honey contains small amounts of enzymes, the most important of which are diastase, glucose oxidase, invertase, acid phosphatase and catalase [39]. The honey enzymes have been the subject of numerous studies with the aim of distinguishing between natural and artificial honey [40]. Specifically, diastase activity was used to determine the botanical origin of honey [41]. Today, however, diastase is mainly used as a measure of honey freshness, as the activity of this enzyme decreases in mature and heated honey. Namely, diastase is a thermolabile enzyme that breaks down starch and is used as an indicator of the quality and freshness of honey, as it determines the degree of damage to honey caused by heating or improper storage at high temperatures [26]. The diastase activity in the examined samples ranged from 8.40 to 29.50 DN, and a statistically significant difference was found between the diastase activity of all organic and conventional honey samples of the corresponding botanical species (Table 1). The obtained results are in accordance with the regulations which prescribe that the diastase activity in honey should be more than 8 DN [1,22,30]. Conventional honey had a higher diastase activity than organic honey of the corresponding botanical origin, with the exception of organic linden honey (Table 1). Studies have shown that diastase activity in honey of different origins was registered in a wide interval from 0.40 to 22.08 DN [29], while Persano Oddo et al. [39] registered diastase activity from 0.00 to 50.0 DN analyzing 499 honey samples of different botanical and geographical origin.

The pH value of honey is an indicator of the possibility of microorganism growth. The optimal pH value for the growth of microorganisms is 7.2–7.4, while the acceptable pH of honey is 3.2–4.5, which is considered to inhibit the growth of microorganisms in honey [42,43]. According to Bogdanov and associates [24], honey is acidic, with a pH value of 3.5–5.5. In the tested samples, the pH values were between 3.30 and 4.95 and a statistically significant difference in pH values was determined between all organic and conventional honey samples of the corresponding botanical species (Table 1). In the case of conventional chestnut honey, the pH value was outside the range that is considered suitable in terms of antimicrobial activity. The pH values are used to distinguish nectar-honey (low pH values–3.5–4.5) and honeydew (high pH values—4.5–6.5; [4,44]), but, according to Bogdanov and co-workers [24], all tested honey samples (organic and conventional) can be classified as acidic, regardless of the production method.

There was no established dependence (*r* = −0.08; Table S3) between the pH value and the results of testing the free acidity of honey. This is in agreement with literature data [45], according to which the free acidity and pH value of honey are not directly dependent due to the buffering effect of acids and minerals present in honey [44]. The free acidity values of the tested samples ranged from 4.0 to 9.0 mmol/kg, where the free acidity values of organic honey samples (4.0–9.0 mmol/kg) were higher than the values for conventional honey (4.0–8.0 mmol/kg) of the corresponding botanical origin (Table 1). Increased acidity may indicate a higher mineral content in honey [45] and may lead to sugar fermentation, resulting in the formation of organic acids, which affect the taste and microbiological stability of honey [4]. Also, the acidity of honey is affected by the presence of lactones, esters and inorganic ions in honey [43], as well as phenolic acids, vitamin C and proteins, which donate hydrogen ions and contribute to the acidity of honey [46]. However, although slightly higher values for free acidity were registered in organic honey samples, they are still far lower than the maximum allowed value (50 meq/kg; [1]) as well as than values in studies by other authors (for example 17.55–31.83 meq/kg, [42]; or 12.0–134.5 meq/kg, [44]; or 6.45–124.20 meq/kg, [29]). The obtained values for free acidity in this study are in agreement with the relatively wide range of these values obtained by Šarić and associates [47]. These authors determined values for free acidity of acacia honey of 5.0–15.1 mmol/kg, of chestnut honey of 6.0–21.7 mmol/kg and of meadow honey of 7.0–37.7 mmol/kg, depending on the three annual seasons (2003–2005), which confirms that the acidity of honey is determined by the season of honey collection [48]. A strong correlation was found between the free acidity value and the electrical conductivity of the samples (*r* = 0.78; Table S3), which is in agreement with studies emphasizing that the electrical conductivity of honey depends on the content of acids, with higher acid contents in honey trigger higher conductivity [31,49].

Total soluble solids in the tested samples were expressed in Brix degrees. Since the degree of Brix corresponds to 1% of sugar [49], it can be concluded that the examined samples had a sugar content ranging between 77.16% and 84.25% (Table 1), whereby the samples of conventional honey showed slightly lower values than the samples of organic honey, except for meadow honey (which had the same values in the conventional and organic samples). Most of the samples analyzed contained soluble solids in the range of 78.77–316.92 °Brix, which was in agreement with Solayman and co-workers [29] giving an overview of the physicochemical characteristics of about 1000 honey samples from all over the world. Slightly lower values than these were registered in linden honey of organic and conventional production (77.50 and 77.16 °Brix, respectively; Table 1). Honey is a concentrated aqueous solution composed mainly of a mixture of fructose and glucose, but it also contains at least 22 other carbohydrates [29]. As sugars are the main constituents of honey, the physical characteristics, as well as the sweetness, are attributed to the sugar’s composition [29].

Honey can be classified by color, a physical property that is very easy to observe and sensory characteristics that are very important to the consumer. The color of honey can range from very light—water white, through amber tones, to very dark, almost black, with possible shades of greenish, light yellow, or reddish [24]. The color of honey can be evaluated by sensory analysis, as well as by physical methods based on visual comparison, using different scales, such as the Pfund-grading [50]. A statistically significant difference in the Pfund values was found for honey color (Table 1) between organic and conventional honey samples of the corresponding botanical species. The Pfund scale values for the tested samples ranged from −8.62 to 126.57 mm Pfund. The color intensity ranged from watery white to dark amber. The most intensely colored honey among the examined samples was organic and conventional chestnut honey, while organic and conventional acacia honey, according to the Pfund scale, was the lightest (Table 1). The color of honey depends on the chemical composition (mineral elements, pollen and phenolic compounds), botanical and geographical origin, as well as on the method of production, agricultural practices, storage temperature and storage time [51,52]. The darkening of honey can occur due to Maillard reactions, fructose caramelization reactions, and reactions to phenolic compounds during honey storage [51,53]. According to Solayman and co-workers [29], darker-colored honey contained more mineral elements compared to lighter-colored honey. In this study, a moderate correlation (*r* = 0.66; Table S3) was found between honey color and the total content of mineral substances. There are conflicting opinions in various studies about the relationship between honey color and the content of phenolic components. For example, Bogdanov and co-workers [26] found that darker honey contains more flavonoids, anthocyanins, tannins and sugar. According to Moniruzzaman et al. [54], a higher Pfund value and color intensity indicate an increased content of phenolic components, in particular flavonoids in honey. On the contrary, Amiot and co-workers [55] pointed out that darker-colored honey contains fewer flavonoids and more phenolic acid derivatives compared to lighter-colored honey. The results obtained in this study showed a strong dependence between the Pfund values and the content of total flavonoids (*r* = 0.88) and hydroxycinnamic acid derivatives (*r* = 0.82), while a moderate dependence was registered between the Pfund values and the content of total phenolic content (*r* = 0.63; Table S3).

### 3.2. Content of Phenolic Compounds

Phenolic compounds are aromatic phytochemicals with important antioxidant activity in honey. They are natural products of secondary plant metabolism and reach honey through honeybees [56]. Their range in honey is very wide; according to some authors, it ranges from 5 to 1300 mg/kg [57,58], while according to others from 20 to 2400 g/100 g of honey [44].

**Figure 1:**
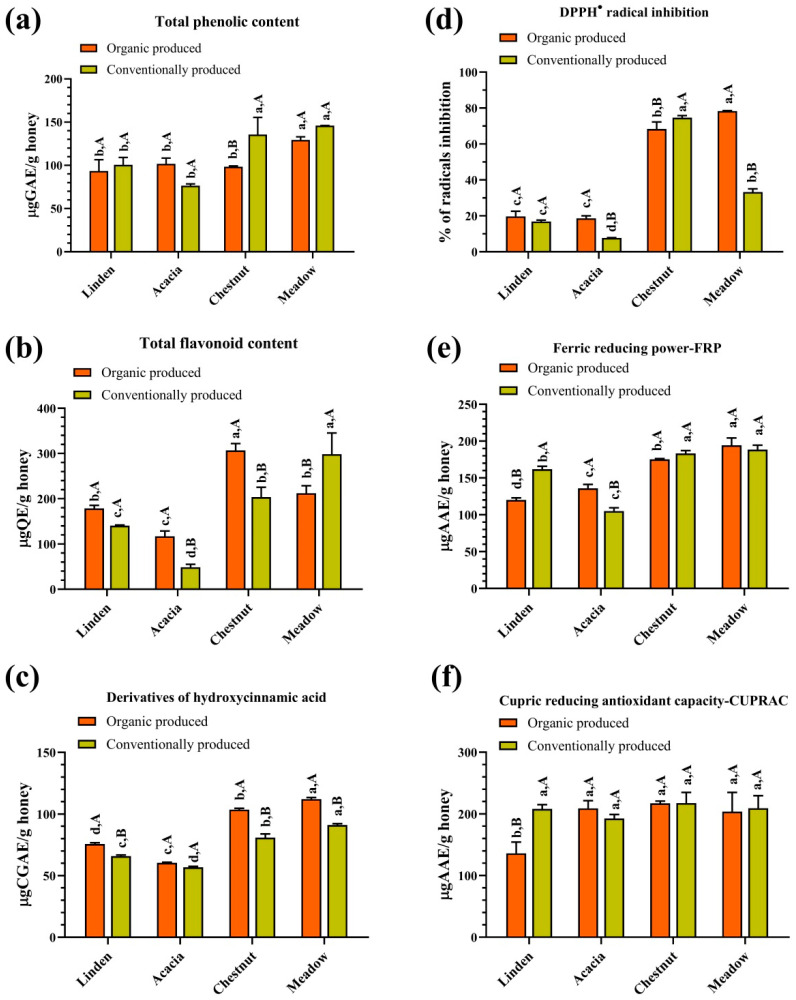
Spectrophotometric assays: (**a**) Total phenolic content; (**b**) Total flavonoid content; (**c**) Total derivatives of hydroxycinnamic acid; Antioxidant assays: (**d**) DPPH radical inhibition activity; (**e**) Ferric reducing power—FRP; (**f**) Cuprac reducing antioxidant capacity—CUPRAC. Lowercase letters indicate comparisons of the honey samples of the different botanical origins produced in the same way (organic or conventional); Uppercase letters indicate comparisons of type production of the same botanical origin honey. Different letters indicate statistically significant differences according to Tukey’s test (*p* < 0.05).

A statistically significant difference was found in the results for total phenolic content between organic and conventional honey samples of the corresponding botanical species, except for organic and conventional linden honey (Figure 1a). The value of total phenolic content in the analyzed samples ranged from 76.5 to 145.9 μgGAE/g, with the highest content of total phenolics found in conventional meadow honey at 145.9 μgGAE/g, while the lowest content of these compounds was recorded in conventional acacia honey at 76.5 μgGAE/g. Comparing the samples analyzed, conventional honey of different botanical species contained more total phenolics than organic honey, except for acacia honey samples (Figure 1a). The values for the total phenolic content determined in this study do not differ significantly from the published results. Acacia honey has been reported to have a phenolic content of 0.51–0.63 mgGAE/g [59] and of 129.16–341.67 mgGAE/kg [60]. Different values for the content of total phenolics have also been published for honey of other botanical origins: for example, for linden honey from 12.30 to 15.03 mgGAE/100 g [61]; for chestnut honey of 0.12 mgGAE/g [62] and in the range of 487–1134 mgGAE/kg [63] and for meadow honey of 21.3 mgGAE/100 g [64], as well as of 265.1 mgGAE/mL [65]. Polak-Śliwińska and Tańska [10] registered a significantly higher content of phenolics in conventional samples of examined honey than in samples produced by organic beekeeping. These results indicate that the total phenolic content may be much more influenced by factors such as geographical origin, time of honey collection, method and duration of storage, and agrometeorological conditions than by botanical origin or type of beekeeping.

Flavonoids are a large family of plant phenolic pigments. More than 90% of honey flavonoids come from propolis, suggesting that flavonoids are more important for the identification of geographical origin than in studies on botanical origin [66]. A statistically significant difference was found in the total flavonoid content between organic and conventional honey samples of the same botanical species. The values for the total flavonoid content ranged from 48.7 to 307.0 μgQE/g (Figure 1b). The values of total flavonoids in the organic honey samples were higher than in conventionally produced honey, with the exception of meadow honey (Figure 1b). The organic chestnut honey sample was the darkest of the honey samples analyzed (Table 1) and had the highest total flavonoid content (307.0 μgQE/g), while the conventional acacia honey sample was the lightest and contained the lowest flavonoid level (48.7 μgQE/g; Figure 1b), which is consistent with the data in the literature that darker honey contains more flavonoids and lighter honey contains less [4,54]. Similar to the data for the total phenolic content in honey, the values for the total flavonoid content also vary widely. For example, for acacia and linden honey, the values for flavonoid content range from unidentified [59,62] to the range of 28.83–113.06 mgQE/kg and 20.92–30.32 mgQE/100 g, respectively [60,67]; for meadow honey 6.14 mgQE/100 g [64] or 13.60 mg catechin equivalents/kg [68]. While studies show that flavonoids are mostly not identified in chestnut honey [69], or they are identified at low levels (1.34–3.76 mgQE/100 g; [70]. In this study, almost ten times higher values for the total flavonoid content were found in some samples of chestnut honey analyzed than in these literature data. The reason for this could be the influence of the different geographical origins of the honey.

The levels of hydroxycinnamic acid derivatives were higher in all organic honey samples than in conventionally produced honey. A statistically significant difference was found in the results for hydroxycinnamic acid derivatives content between organic and conventional honey samples of the corresponding botanical species (Figure 1c). The values of hydroxycinnamic acid derivatives ranged from 56.7 to 112.1 μgCGAE/g. The highest content of hydroxycinnamic acid derivatives was found in organic meadow honey at 112.1 μgCGAE/g, while the lowest content of these compounds was in conventional acacia honey at 56.7 μgCGAE/g (Figure 1c). This group of compounds is synthesized by plants via the shikimate metabolic pathway [71] and enters honey via bees, which can contribute significantly to the nutritional value of honey (for example, anticancer, antimicrobial and antioxidant; [72].

### 3.3. UHPLC Profile of Phenolic Components

A detailed analysis of the profile of bioactive compounds of honey samples should provide several useful pieces of information, such as (1) confirmation of similarities/differences between organically and conventionally produced honey samples; (2) identification of potential markers for the botanical origin of the honey and (3) evaluation of the functional capacity of the analyzed honey. A total of 38 phenolic compounds (in negative ionization mode) and 4 phenylamides (in positive ionization mode) were identified in all analyzed honey samples by UHPLC Q-ToF MS analysis. All compounds were identified based on the exact *m*/*z* mass of the molecular ions and the typical MS fragments, as listed in Table 2, while the results of their quantification (µg/g honey) are shown in Table 3. The total content of identified phenolic compounds ranged from 55.62 (conventional chestnut honey) to 1216.91 (conventional meadow honey) µg/g honey. The quantification confirmed that the total content of the identified phenolic compounds originated primarily from propolis-derived flavonoid aglycones (PDFAs) such as pinocembrin, chrysin, pinobanksin and galangin [73] (Table 3), which is consistent with the results of other studies [74,75,76,77]. These PDFAs were confirmed in all honey samples (with the exception of galangin in conventional chestnut honey), and their amounts are obviously closely related to the presence and content of propolis in honey. The lowest content of identified total phenolics and PDFAs was found for both chestnut honey (organic and conventional), while their amount varied in the other analyzed honey samples. Furthermore, the total amount of identified phenolics and PDFAs was higher in organic linden, acacia and chestnut honey than in conventional honey, which was not the case for meadow honey. In view of these results, the presence and content of PDFAs are not representative markers for confirming the botanical origin and selecting the production method of honey.

Other phenolic compounds were found selectively and may be potential indicators of the botanical origin of the analyzed honey samples. Among the phenolic acid derivatives, caffeic acid derivatives were the most numerous, but their presence in the honey samples was selective and was below the limit of quantification for most derivatives (<LOQ). The caffeic acid derivatives were mainly detected in acacia honey as well as in conventional meadow honey. In contrast, these derivatives were completely absent in chestnut and organic meadow honey. The clear differences in the profiles of meadow honey may be due to the presence of different polyfloral pollen grains in the composition of this honey. The detected prenyl caffeate and caffeic acid phenethyl esters (CAPE) are the most common compounds found in propolis [78,79,80], and their content probably depends on the presence of propolis in honey. Special attention should be paid to ethyl caffeate, which was only detected in linden honey. Benzoic acid, coumaric acid and esculetin were selectively detected in small amounts or traces in the acacia, chestnut and meadow honey examined. Abscisic acid was quantified in both acacia and linden honey as well as in organic chestnut honey. This result is consistent with other studies that have identified abscisic acid as a potential marker for linden and acacia honey [74,76,77].

The phenylamides identified in the honey samples originate from pollen and may be potential botanical origin markers. For example, dicoumaroyl spermidine was only detected in meadow honey, while dicoumaroyl caffeoyl spermidine was only confirmed in chestnut honey. However, further investigations are needed as different coumaroyl derivatives are present in most cases in different bee-collected pollen samples [14,81,82]. Tri-coumaroyl spermidine was found in acacia, chestnut and meadow honey, while phenylamides were not detected in linden honey.

Among the flavonols (with the exception of galangin), significant amounts of quercetin dimethyl ether (compound 24, Table 3) were detected in organic linden and acacia honey and isorhamnetin in both chestnut kinds of honey. Two kaempferol rhamnosides (compounds 25 and 26, Table 3) were only detected in organic acacia honey. These compounds originated from the nectar of acacia flowers and are typical markers for this honey [83]. Pinobanksin esters are characteristic compounds from propolis, and their content probably depends on the proportion of propolis in the honey. These pinobanksin derivatives are easy to detect as they show a typical fragmentation with two main fragments at 271 *m*/*z* (deprotonated pinobanksin) and 253 *m*/*z* (-H_2_O) (Table 2). Apart from pinobanksin, a significant content of pinobanksin-5-methyl ether (compound 28) was detected in all analyzed honey, except in organic chestnut honey. Other detected pinobanksin derivatives (except pinobanksin-3-O-pentanoates) were only detected in conventional meadow honey. Acacetin was only not detected in conventional chestnut honey, while its content in the other honey samples varied between 5.00 and 75.98 µg/g. Apigenin and luteolin-methyl-ether were found in both acacia and meadow honey, while genkwanin was only confirmed in acacia honey. Sakuranetin was quantified in organic linden and meadow honey.

### 3.4. Antioxidant Properties

Many of the honey phenolic compounds are known to have antioxidant activity [70,84,85]. Many authors reported that the content of certain phenolic compounds has a strong linear correlation with the antioxidant activity of honey [84,85,86,87]. This study showed that antioxidant activity is not strongly dependent on the content of certain phenolic compounds in honey. Indeed, depending on the method used to determine the antioxidant properties of the honey samples tested, a weak, medium, and strong correlation was found (Table S3). This indicates that the antioxidant properties of honey are not entirely due to the phenolic compounds alone. Although individual phenolics may have considerable antioxidant potential, there may be antagonistic or synergistic interactions between non-phenolic and phenolic compounds. The other constituents (e.g., carotenoids, α-tocopherol, ascorbic acid, organic acids, amino acids and proteins, enzymes (glucose-oxidase, catalase), minerals or Maillard reaction products (melanoidins) that are present in raw honey [88,89] could contribute to the overall antioxidant activity. For example, Meda and co-workers [90] found a higher correlation between radical scavenging activity and proline content than with total phenolic compounds. Therefore, in complex food matrices, where there are many potential antioxidants with different mechanisms of action, it is recommended to use several different methods to determine the antioxidant capacity [91]. Three methods were used to evaluate the antioxidant activity of the tested honey samples: DPPH^●^ radical scavenging capacity (DPPH), ferric-reducing power (FRP) assay and cupric-reducing antioxidant capacity (CUPRAC).

The antiradical activity of the honey samples was estimated using the DPPH assay, and stronger activities were observed in organically produced honey than in conventionally produced honey (18.62–78.35 and 7.72–74.68% radical inhibition, respectively; Figure 1d). The chestnut honey samples deviated from this, but no statistically significant differences were observed between the degree of radical inhibition of organic and conventional chestnut honey. The obtained results for radical inhibition differed significantly for the honey of different botanical origins, with the lighter honey (linden and acacia, from organic and conventional beekeeping) showing significantly lower values (7.72–19.72% radical inhibition; Figure 1d). The strong correlation between the hydroxycinnamic acid derivatives content (*r* = 0.83; Table S3) and the values obtained by the DPPH assay suggests that these phenolic acids play an important role in the inhibition of DPPH^●^ radicals. The ratio obtained between the total phenolic compounds and the degree of radical inactivation (Table S3) was in agreement with the results of [92], who also emphasized the mean correlation between these parameters in the study of 32 honey of different floral origin. The DPPH assay is frequently used to test the antioxidant properties of honey and is mainly used to determine phenolic antioxidants soluble in organic media [93]. Wilczyńska [92] pointed out in her study that linden honey has a radical inhibition of 63.64% and acacia honey of 35.90%, while Predescu and co-workers [94] registered a degree of radical inhibition of 45.12% in meadow honey. Significantly lower values for the degree of free radical inhibition in linden and acacia were registered in the examined honey samples (Figure 1d) compared to the results of Wilczyńska’s [92]. This could be due to the different geographical origins of the honey samples.

The FRP assay shows the ability of antioxidants to reduce Fe^+3^ ions, with a higher value indicating a stronger reducing power [93]. Even when using the FRP assay, no general differences were found between organically and conventionally produced honey (105.1–194.4 mgAAE/g; Figure 1e). The darker-colored honey (organically and conventionally produced chestnut and meadow honey; Figure 1e) showed the highest ability to reduce Fe^+3^ ions. The lightest acacia honey showed the lowest values for antioxidant activity, as determined by the FRP assay. Since strong correlations were found between the results of the FRP assay and the content of phenolic compounds (TPC, TFC, DHCA; Table S3), it can be concluded that phenolic compounds play an important role in the reduction of Fe^+3^ ions. In the literature data available to us, there are no studies of the antioxidant properties of honey using the FRP assay.

The results of the antioxidant activity test using the CUPRAC method show that both groups of honey tested (organic and conventional) show a high ability to reduce Cu^+2^ ions (136.1–217.5 mgAAE/g) with the lowest value recorded for the organic meadow honey (Figure 1f). The highest antioxidant activity was found for chestnut honey, with no significant differences between chestnut honey samples from organic and conventional beekeeping (216.9 and 217.5 mgAAE/g, respectively; Figure 1f). In fact, the results obtained with the CUPRAC assay were not statistically different according to the production method (organic/conventional) or botanical origin, with the exception of the sample of organically produced linden honey. No significant correlation was found between the results of the CUPRAC assay and the content of the phenolic compounds analyzed (Table S3). This indicates the ability of the non-phenolic constituents in honey to reduce Cu^+2^ ions, which should be interesting to examine in future work. In the literature data available to us, there is limited data on the antioxidant properties of honey obtained using the CUPRAC assays [95]. For example, the antioxidant activity of chestnut honey was registered from 11.00 to 97.07 mmol Trolox/100 g using this method [96].

### 3.5. Mineral Composition

The mineral content of nectar honey is generally low and ranges between 0.02% and 0.3% [97]. The mineral composition of honey is influenced by several factors, such as soil and climatic conditions, the chemical composition of the nectar (which varies according to the different botanical sources) and beekeeping techniques [29]. The most abundant mineral elements in the samples analyzed were potassium (2225.56 μg/g), phosphorus (923.92–795.40 μg/g) and calcium (15.70–240.60 μg/g), with the greatest differences between samples in calcium content (Table 4). In addition to these macroelements, significant content of other macroelements, such as magnesium, sodium and sulfur, was found in all examined samples (Table 4), which is consistent with literature data [29,98]. Potassium is the most abundant element in honey, accounting for one-third of the total mineral composition, which may be a consequence of its rapid secretion by the nectaries, and the potassium content can be more than ten times higher than the content of other macroelements in honey [98]. Less abundant are the elements iron (0.91–5.44 μg/g), manganese (0.09–6.60 μg/g) and zinc (0.22–3.75 μg/g), which belong to the group of microelements (Table 4). Of the toxic elements in the analyzed samples, the presence of boron (1.71–7.54 μg/g) and aluminum (0.99–4.31 μg/g) was detected in the highest concentration, while the presence of toxic elements such as lead and arsenic was not recorded, and lithium was practically in traces (0.008–0.017 μg/g; Table 4). The presence of toxic elements in honey is actually a consequence and indicator of environmental pollution [29]. Therefore, the mineral composition of honey is considered an indicator of environmental pollution [99]. As the mineral composition of honey is a direct result of its presence in the environment, published studies have also found wide variation in its presence in honey. For example, the magnesium content of honey was found to range from 2.18 to 563.72 mg/kg, the iron content from 0.41 to 224.00 mg/kg, or the zinc content from 0.05 to 17.30 mg/kg of honey [29,100].

### 3.6. Raman Spectroscopy Analysis with PCA Analysis

**Figure 2:**
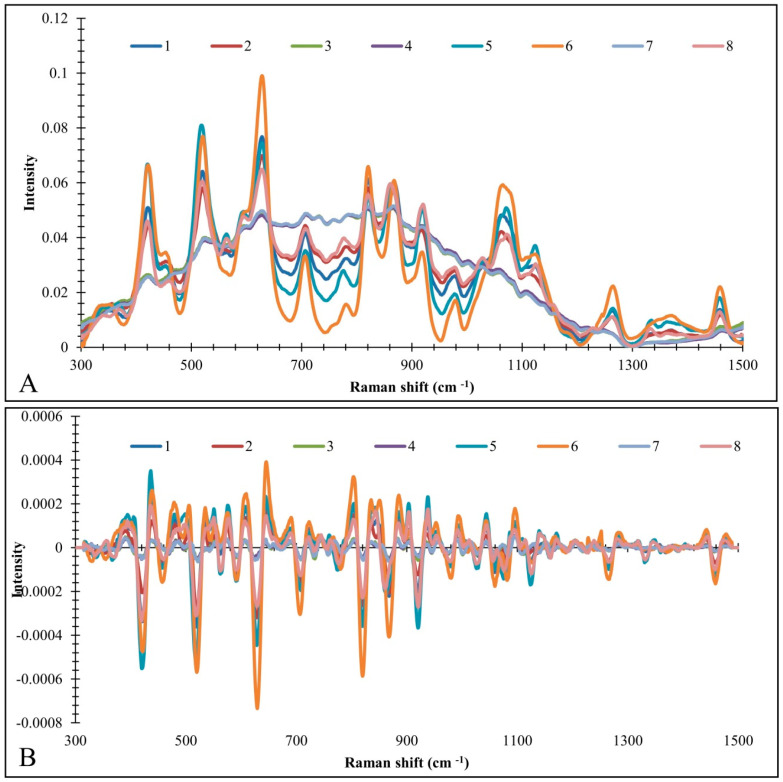
Raw spectra (**A**) and pre-processed spectra (baseline correction + normalization + 2nd order derivative and smoothing (**B**). Legend: 1—linden organic; 2—accacia organic; 3—chestnut organic; 4—meadow organic; 5—linden conventional; 6—accacia conventional; 7—chestnut conventional; 8-meadow conventional.

The recorded Raman spectra of different honey samples were presented in the fingerprint region, i.e., between 300 and 1500 cm^−1^ (Figure 2A). The characteristic band in all honey samples is identified at 353 cm^−1^ and can be assigned to the δ(C-C-C) ring vibration of carbon hydrates [101]. The bands recorded at 422 cm^−1^, 519 cm^−1^ and 628 cm^−1^ [102] could be assigned to δ(C-C-O) [103], δ(C-C-C) carbohydrates [102,103], and ring deformation, respectively [102,104]. The band at 709 cm^−1^ contributes to ν(C-O), ν(C-C-C) and δ(O-C-O) [102]. The bands at 821 cm^−1^, 867 cm^−1^ and 920 cm^−1^ contribute to ν(C-O-H) [102,105], δ(C-H) [102,106], and δ(C-O-H) [102,107], respectively. Intensive bands were also identified at 1061 cm^−1^ and 1124 cm^−1^. The first may be attributed to ν(C-C), ν(C-O) and δ(C-O-H) carbohydrates [106]. The second band is most likely linked to ν(C-O) and δ(C-O-H) chemical bonds [102,103].

**Figure 3:**
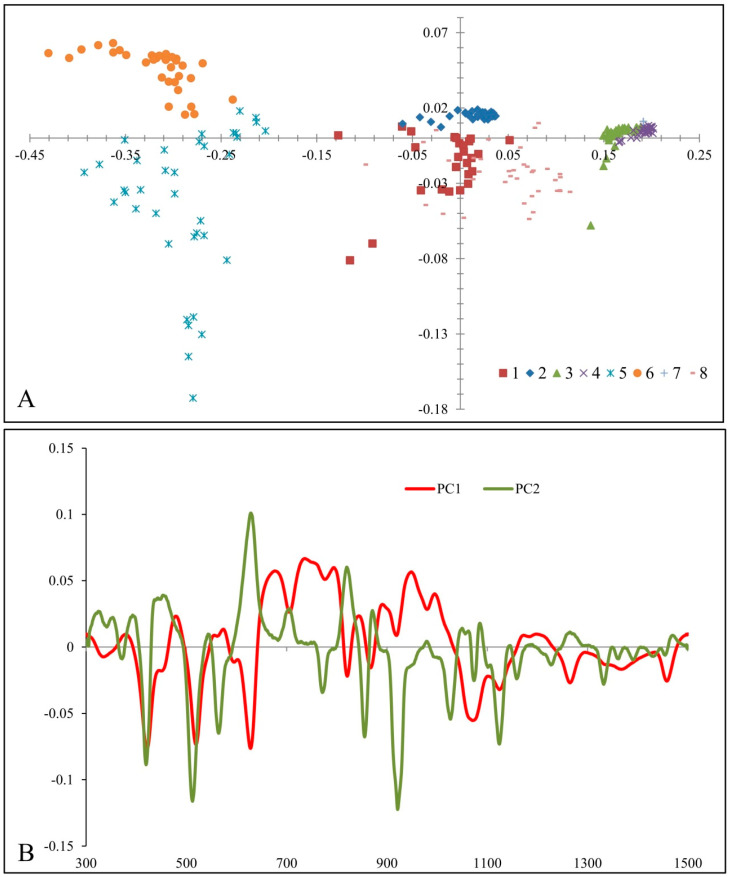
PCA score plot (**A**) and Loading plot (**B**). Legend: 1—linden organic; 2—accacia organic; 3—chestnut organic; 4—meadow organic; 5—linden conventional; 6—accacia conventional; 7—chestnut conventional; 8—meadow conventional.

The results of the PCA are presented by score and loading plots (Figure 3). The score plot shows a clear tendency to group the different honey samples (Figure 3A). According to the PC1 axis, samples 5 and 6 and samples 3, 4 and 7 were grouped jointly. The results of the loading plot indicate that samples 5 and 6 have been separated as one distinct class due to the negative results at ~422, ~519 and ~628 cm^−1^. Samples 3, 4 and 7 were grouped in one separate cluster due to the positive loading values of PC1 at ~820 cm^−1^. On the other hand, samples 1 and 5, in addition to samples 2 and 6, were grouped together due to the PC2 values. The results of the loading plot indicate that strong bands at ~628, ~821, and ~867 cm^−1^ were responsible for the grouping of samples 2 and 6, whereas bands at ~422, ~920 cm^−1^ determined the separation of samples 1 and 5 into one distinct class (Figure 2B).

The results of the classification of different honey samples are shown in Tables S4 and S5. In both models tested, the 5 PCs were used, explaining 99% of the total variability. In the first case, using the model based on 8 classes (corresponding with 8 studied honey samples), the accuracy was between 83.33–100.00% (96.88% in total) (Table S4). In the second case, using 4 classes based on the botanical origin of the studied honey samples, the model accuracy ranged between 84.72 and 98.61% (92.36% in total). The graphical representation of the discrimination results is presented in Figures S1 and S2.

Madgas et al. [108] used Raman spectroscopy and chemometrics to classify different honey types. Based on Soft Independent Modeling of Class Analogy (SIMCA), their results showed high percentage accuracy for acacia (100%), chestnut (100%) and linden (83%). Oroian and Ropciuc [101] applied Raman spectroscopy and linear discriminant analysis (LDA) to determine the botanical origin of different honey samples. Accordingly, honeydew samples were correctly classified in 95% of the studied samples, while in the case of Acacia honey, the accuracy was 90%.

To the best of our knowledge, there is no work based on the application of Raman spectroscopy and chemometrics in distinguishing the honey samples originating from different production sites, which were simultaneously distinct production systems (organic and conventional). Our results indicate that Raman spectroscopy associated with the appropriate chemometric modeling has successfully classified different honey samples. However, fine differences in spectra of the same honey types obtained from the different production systems are most likely the consequence of a specific chemical composition of a sample reflecting peculiarities of different honey-bee collecting sites -locations (bee pastures), rather than production systems (organic versus conventional) since organic (certified) and conventional honey production sites have to be sufficiently distant. Finally, there was no typical Raman band specifically corresponding to either organic or conventional honey samples. However, Raman spectroscopy showed very high validity for the classification of different honey samples based on their botanical origin.

### 3.7. Sensory Analysis

**Figure 4:**
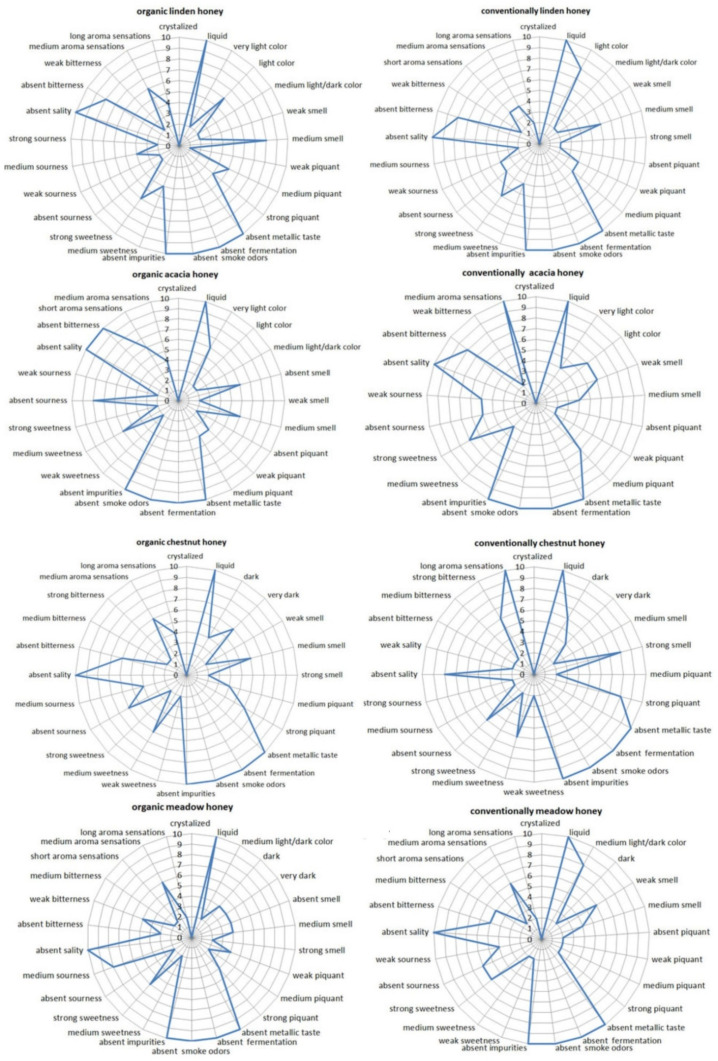
The result of the sensory evaluation of the tested honey samples by trained evaluators (n = 2 × 10).

Sensory analysis of honey is a fast and practical way to obtain information about the quality of honey and is often used to determine the price of honey [109]. It can also detect undesirable characteristics that are not reached by routine analysis, such as metallic taste, fermentation, smoky odor, or the presence of impurities [17]. Honey is characterized by specific sensory properties due to the large number of components that come from both the nectar and the bees themselves. When comparing organic and conventional honey samples using the hedonic rating scale, higher scores were obtained for the overall sensory acceptability of samples from organic beekeeping (except for linden honey, where the scores are very close to each other; Table 1). Organic acacia honey scored the highest for overall acceptability (7.7), while conventional chestnut honey scored the lowest (4.1). The reason for the low overall acceptability rating of conventional chestnut honey could be due to experts’ assessment of spiciness and bitterness (Figure 4). Six out of ten expert evaluators stated that the smell of the organic acacia honey was not present, while 8 out of 10 evaluators declared that the conventional chestnut honey had a strong smell (Figure 4). According to Bogdanov [13], over 600 aromatic compounds were detected in different types of honey. Most of the volatile compounds come from the flower of the plant and certain monofloral honey. Certain volatiles are found exclusively in certain types of honey and are used to accurately test the botanical origin of honey [26]. The evaluators stated that chestnut and meadow honey were the darkest in color, corresponding to dark and light amber shades of the Pfund scale (Figure 4; Table 1). None of the samples tested were found to have undesirable characteristics, such as fermentation, metallic taste, smoky odors or the presence of impurities (Figure 4). The sensory characteristics of the tested honey samples clearly depended on the botanical origin but not on organic or conventional beekeeping.

## 4. Conclusions

Although statistically significant differences in physicochemical parameters (specific optical rotation, electrical conductivity, moisture, diastase activity, free acidity, pH) were found between all samples of organic and conventional honey of the corresponding botanical species, no general trend in the parameters depending on the beekeeping method can be established. These slight differences within the same botanical origin may be due to different geographical origins, although all samples are from the Balkans. The mineral composition, total phenolic and total flavonoid contents, profile of phenolic compounds and antioxidant properties significantly depended on the botanical origin of the honey and not on the beekeeping method. Organic honey samples were only richer in hydroxycinnamic acid derivatives. In the phenolic profile of the analyzed samples, 38 phenolic compounds and 4 phenylamides were identified, with the largest proportion contributed in most samples coming from propolis-derived flavonoid aglycones (pinocembrin, chrysin, pinobanksin and galangin). Raman spectroscopy did not show the differentiation of honey according to the beekeeping method (organic/conventional) but showed the botanical origin. No clear differences were observed between the sensory properties of honey samples from organic and conventional beekeeping. The PCA analysis did not reveal any general differences between organic and conventional honey samples. The general conclusion is that the physiochemical, nutritional and sensory characteristics do not depend significantly on the method of honey production (organic or conventional beekeeping) but much more on the botanical origin. However, as legal regulations prescribe the absence of pesticides and other anti-nutritive components in organic honey, in the future, differences in the content of these components between samples obtained from organic and conventional beekeeping could be examined. In this way, a complete “picture” of both the quality and the safety of the tested samples would be obtained. Also, in future studies, a larger number of samples should be considered in order to confirm the obtained statistical models.

## Figures and Tables

**Table 1:** Physicochemical characteristics of tested honey samples, their classification by color and overall sensory acceptability ^a^.

Physicocheical Characteristics								
Honey Samples		Specific Optical Rotation [α]_D_^20^	Electrical Conductivity (mS/cm)	Moisture (%)	pH	Free Acidity (mmol/kg)	Diastasis (DN)	Soluble Solids (°Brix)
organic produced	linden	−1.35 ^d^	0.80 ^c^	18.39 ^a^	3.68 ^e^	7.0 ^c^	29.50 ^a^	77.50 ^e^
	acacia	−1.83 ^c^	0.16 ^h^	14.43 ^e^	3.41 ^f^	4.0 ^e^	8.40 ^e^	84.25 ^a^
	chestnut	−0.81 ^f^	0.95 ^b^	16.00 ^d^	4.34 ^b^	6.5 ^cd^	16.20 ^d^	82.66 ^b^
	meadow	−1.00 ^e^	0.98 ^a^	17.50 ^c^	4.11 ^c^	9.0 ^a^	16.30 ^d^	81.00 ^c^
conventionally produced	linden	−1.00 ^e^	0.72 ^d^	18.29 ^a^	3.83 ^d^	6.0 ^d^	17.20 ^c^	77.16 ^e^
	acacia	−2.60 ^a^	0.19 ^f^	18.00 ^b^	3.30 ^g^	4.0 ^e^	18.30 ^c^	78.83 ^d^
	chestnut	−1.25 ^d^	0.81 ^c^	18.11 ^b^	4.95 ^a^	4.0 ^e^	29.20 ^a^	78.50 ^d^
	meadow	−2.10 ^b^	0.43 ^e^	17.60 ^c^	3.23 ^h^	8.0 ^b^	25.90 ^b^	81.00 ^c^
Pooled std		0.06	0.003	0.07	0.02	0.25	0.44	0.20
Classification by colour and overall sensory acceptability								
honey samples		mm Pfund *	colour name		optical density		overall acceptability **	
organic produced	linden	29.64 ^e^	white		0.378		6.2 ^a^	
	acacia	1.04 ^h^	water white		0.0945		7.7 ^b^	
	chestnut	126.57 ^a^	dark amber		-		7.0 ^c^	
	meadow	76.06 ^d^	light amber		1.389		7.2 ^d^	
conventionally produced	linden	17.00 ^f^	extra white		0.189		6.5 ^af^	
	acacia	−8.62 ^g^	water white		0.0945		7.2 ^d^	
	chestnut	112.08 ^b^	amber		3.008		4.1 ^e^	
	meadow	84.60 ^c^	light amber		1.389		6.7 ^f^	
Pooled std		0.56	/		/		0.03	

^a^ Means in the same column with different letters are a significant difference according to the *t*-test (*p* < 0.05). * Pfund Scale—millimeters of the Pfund scale. ** Overall acceptability was the result of the “hedonic scale” of two replicates; n = 2 × 60). Results are shown as mean and pooled standard deviation (Pooled std) of three replicates (unless otherwise specified).

**Table 2:** Identification and characterization of phenolic compounds in various organic and conventional produced honey samples, using UHPLC Q-ToF MS analysis. Identified compounds, expected retention time (RT), molecular formula, calculated mass, *m*/*z* exact mass and MS fragments are presented in Table.

No.	RT	Compound Name	Formula	Calculated Mass	*m*/*z* Exact Mass	mDa	MS Fragments
Phenolic acid and derivatives							
1	6.25	Benzoic acid	C_7_H_5_O_2_^−^	121.029	121.0281	−0.85	/
2	7.41	Coumaric acid	C_9_H_7_O_3_^−^	163.0395	163.0385	−1.02	119.0489(100), 120.052(11), 117.0328(8)
3	6.50	Esculetin	C_9_H_5_O_4_^−^	177.0188	177.0176	−1.18	135.043(100), 134.0352(77), 105.033(16), 133.0277(12), 117.0327(9), 121.0276(5), 149.0223(4)
4	6.58	Caffeic acid	C_9_H_7_O_4_^−^	179.0344	179.0341	−0.33	135.0425(100), 134.034(80), 107.0486(12), 117.0318(11)
5	9.87	Ethyl caffeate	C_11_H_11_O_4_^−^	207.0657	207.0654	−0.33	133.0273(100), 135.0429(76), 134.0342(40), 161.0222(21), 179.0365(2)
6	12.00	Caffeic acid prenyl ester (Prenyl caffeate)	C_14_H_15_O_4_^−^	247.097	247.0961	−0.93	135.0437(100), 133.028(47), 134.0349(31), 161.0225(26), 179.0331(8)
7	12.11	Caffeic acid benzyl ester	C_16_H_13_O_4_^−^	269.0814	269.0803	−1.08	134.035(100), 133.0272(79), 161.0232(20), 135.0378(4), 106.0403(4)
8	12.51	Caffeic acid phenethyl ester (CAPE)	C_17_H_15_O_4_^−^	283.097	283.0962	−0.83	135.0431(100), 161.0229(34), 133.0279(29), 134.0354(23), 179.0331(14)
9	13.04	Caffeic acid cinnamyl ester	C_18_H_15_O_4_^−^	295.097	295.0961	−0.93	134.0354(100), 133.0278(44), 135.0386(11), 106.0413(5), 161.0218(3)
10	5.80	Caffeic acid hexoside is. I	C_15_H_17_O_9_^−^	341.0873	341.087	−0.26	161.0229(100), 135.043(87), 179.0333(41), 133.0274(15), 134.0354(9)
11	6.37	Caffeic acid hexoside is. II	C_15_H_17_O_9_^−^	341.0873	341.087	−0.26	135.0434(100), 179.033(72), 161.0227(54), 134.0352(7)
Non-phenolic compounds							
12	9.47	Abscisic acid	C_15_H_19_O_4_^−^	263.1283	263.1277	−0.63	203.1064(100), 204.1124(60), 122.035(58), 153.0901(43), 136.0512(41), 189.0899(40), 137.0577(29), 164.0811(20), 138.0666(38), 219.1368(14)
Phenolic acid amides (Phenylamides)							
13	7.74	Di-coumaroyl spermidine	C_25_H_32_N_3_O_4_^+^	438.2393	438.2393	0.02	204.101(100), 147.0435(99), 292.2015(31), 205.1047(15), 275.175(11), 218.117(11), 293.2039(7), 438.2371(6), 119.0491(5)
14	9.91	Tri-coumaroyl spermidine	C_34_H_38_N_3_O_6_^+^	584.2761	584.2763	0.24	438.2382(100), 204.1017(42), 439.2411(35), 147.0439(35), 292.2014(31), 275.1753(16), 420.2271(15), 4212235(9), 293.204(7), 205.1046(7), 119.0494(3)
15	9.75	Dicoumaroyl caffeoyl spermidine	C_34_H_38_N_3_O_7_^+^	600.271	600.2715	0.52	438.2382(100), 204.1011(44), 439.2407(35), 454.2333(26), 292.2005(25), 147.043(16), 420.2272(11), 455.2364(11), 275.1767(9), 163.0393(7), 293.205(6)
16	10.42	Tetra-coumaroyl spermidine	C_46_H_51_N_4_O_8_^+^	787.3707	787.3693	−1.39	641.3327(100), 642.3369(57), 643.339(15), 275.1745(13), 623.3225(8), 204.1021(9), 147.0435(5), 478.2727(4), 494.3009(4)
Flavonoids and derivatives							
Flavonol aglycones and glycosides							
17	12.37	Galangin	C_15_H_9_O_5_^−^	269.045	269.045	0	269.0439(100), 169.0647(19), 171.0438(17), 213.0539(14), 143.0489(13), 223.0384(11), 195.0438(10), 197.0591(9), 211.0386(9), 227.0336(7), 269.0436(8)
18	12.25	Galangin-methyl-ether	C_16_H_11_O_5_^−^	283.0606	283.0605	−0.15	268.0356(100), 269.0372(23), 240.0404(9), 151.0017(7), 239.0333(7), 117.0332(7), 164.0091(4), 211.0392(4)
19	11.37	Kaempferide	C_16_H_11_O_6_^−^	299.0556	299.0551	−0.46	284.0306(100), 285.0333(21), 256.0355(9), 133.0277(5), 299.0501(5), 255.0296(2), 257.0433(2), 151.0015(4), 107.0141(4)
20	9.68	Quercetin	C_15_H_9_O_7_^−^	301.0348	301.0352	0.37	151.0016(100), 121.0273(45), 107.0114(39), 152.0041(12), 178.9955(9), 149.0223(9), 285.0398(7), 257.0645(5), 243.0235(5)
21	10.49	Isorhamnetin	C_16_H_11_O_7_^−^	315.0505	315.0497	−0.78	300.0245(100), 109.9994(52), 165.989(49), 255.0283(33), 243.0272(26), 271.0222(22), 301.0296(20)
22	11.39	Rhamnetin	C_16_H_11_O_7_^−^	315.0505	315.0497	−0.78	165.0176(100), 121.0278(62), 300.0261(22), 151.0022(11), 272.0313(5), 271.0263(5)
23	10.95	Quercetin-dimethyl-ether is. I	C_17_H_13_O_7_^−^	329.0661	329.0654	−0.73	271.0224(100), 299.017(97), 243.0281(82), 257.0448(24), 300.0202(22)
24	11.73	Quercetin-dimethyl-ether is. II	C_17_H_13_O_7_^−^	329.0661	329.0659	−0.23	299.0168(100), 271.0234(42), 300.0212(19), 314.0415(12), 301.0235(3), 227.0336(2), 243.0289 (3)
25	9.60	Kaempferol-3-*O*-rhamnoside	C_21_H_19_O_10_^−^	431.0978	431.0968	−1.02	285.0374(100), 284.0306(61), 151.0012(45), 257.0426(34), 431.0957(13), 229.0459(2), 213.0526(3)
26	7.64	Kaempferol 3-*O*-(6″-rhamnosyl)-hexoside-7-O-rhamnoside	C_33_H_39_O_19_^−^	739.2086	739.2065	−2.05	593.1479(100), 594.1509(38), 739.2064(13), 285.038(12), 284.0294(10)
Flavanonol aglycones and derivatives							
27	10.41	Pinobanksin	C_15_H_11_O_5_^−^	271.0606	271.0612	0.55	197.059(100), 125.0232(74), 253.0493(67), 161.0595(61), 107.0126(50),151.0032(32), 271.0596(31), 124.0151(29), 181.0643(16), 225.0541(22), 209.0587(14), 254.052(15)
28	9.80	Pinobanksin-5-methyl-ether	C_16_H_13_O_5_^−^	285.0763	285.0763	0.00	252.0411(100), 138.0306(57), 224.0459(55), 241.0493(32), 253.0447(24), 195.0443(18), 213.054(17), 165.0168(14)
29	13.30	Pinobanksin-3-*O*-propionate	C_18_H_15_O_6_^−^	327.0869	327.0858	−1.06	253.0487(100), 254.0516(21), 209.0589(6), 197.0582(6), 107.012(4), 271.0579(3), 255.054(3), 185.0578(2), 225.0533(2)
30	13.78	Pinobanksin derivative	C_19_H_15_O_6_^−^	339.0869	339.0866	−0.26	253.0480(100), 254.0506(19), 197.0587(7), 209.0585(6), 143.0481(5), 107.0119(4), 255.0552(2)
31	14.16	Pinobanksin-3-*O*-butyrate	C_19_H_17_O_6_^−^	341.1025	341.1017	−0.81	253.0486(100), 254.0523(19), 197.059(5), 209.0592(4), 143.0485(3), 107.0121(3), 255.0549(3), 271.0594(2)
32	14.19	Pinobanksin-3-*O*-pentanoate is. I	C_20_H_17_O_6_^−^	353.1025	353.1009	−1.61	253.0491(100), 254.0517(22), 197.0592(5), 209.0589(5), 143.0487(4), 255.0536(3), 107.0126(3), 185.0587(2)
33	14.90	Pinobanksin-3-*O*-pentanoate is. II	C_20_H_19_O_6_^−^	355.1182	355.1175	−0.66	253.0487(100), 254.0524(19), 197.0593(5), 209.059(49, 143.0483(3), 255.0541(3), 107.0123(2), 185.0587(1)
34	15.61	Pinobanksin-3-*O*-hexanoate	C_21_H_21_O_6_^−^	369.1338	369.1324	−1.41	253.0484(100), 254.0514(18), 197.0579(5), 271.0605(3), 209.0601(3), 143.0464(2)
Flavone aglycones							
35	12.10	Chrysin	C_15_H_9_O_4_^−^	253.0501	253.0502	0.12	253.049(100), 143.0486(68), 107.0127(47), 145.0285(24), 151.0024(24), 119.0488(23), 209.0593(20), 171.0439(16), 185.0594(14), 213.0541(12)
36	10.32	Apigenin	C_15_H_9_O_5_^−^	269.045	269.0446	−0.4	117.0324(100), 151.0013(41), 107.0117(37), 269.0435(28), 149.0229(23), 197.0584(15), 225.0526(13)
37	11.18	Genkwanin	C_16_H_11_O_5_^−^	283.0606	283.0609	0.25	211.0383(100), 239.0329(59), 212.0414(16), 240.0375(14), 167.048(3), 268.0345(3), 283.0589(3)
38	12.71	Acacetin	C_16_H_11_O_5_^−^	283.0606	283.0605	−0.15	211.0384(100), 239.0331(66), 212.0421(17), 240.0382(15), 268.0358(5), 167.0485(3), 241.0404(2), 213.0444(2)
39	10.78	Luteolin-methyl-ether	C_16_H_11_O_6_^−^	299.0556	299.0547	−0.86	255.0281(100), 227.0331(79), 284.0303(24), 257.0339(3), 211.0373(3), 132.0194(2), 107.0116(1)
Flvanone aglycones							
40	12.27	Pinocembrin	C_15_H_11_O_4_^−^	255.0657	255.0666	0.87	107.0132(100), 171.0445(93), 151.0028(88), 145.065(76), 213.055(62), 255.0652(43), 185.0596(38), 211.0748(18)
41	13.11	Pinostrobin	C_16_H_13_O_4_^−^	269.0814	269.0807	−0.68	121.0275(100), 165.0177(76), 269.0785(58), 227.0688(57), 183.0791(37), 171.0434(45), 150.0311(30)
42	12.18	Sakuranetin	C_16_H_13_O_5_^−^	285.0763	285.0763	0.00	164.0098(100), 136.0146(75),108.0201(41), 151.0021(30), 107.0122(25),243.0643(16), 285.0743(15), 270.0441(12), 165.0141(11), 201.0532(7), 227.0322(5)

Abbreviations: “is.”—isomer.

**Table 3:** Quantification of phenolic compounds (µg/g) identified in various organic and conventional produced honey samples, using UHPLC Q-ToF MS.

No.	Compound Name	Honey (µg/g honey)							
		Organic Produced				Conventional Produced			
		Linden	Acacia	Chestnut	Meadow	Linden	Acacia	Chestnut	Meadow
Phenolic acid and derivatives									
1	Benzoic acid ^b^	-	-	-	-	-	<LOQ	-	-
2	Coumaric acid ^b^	-	2.33	-	<LOQ	-	-	<LOQ	1.48
3	Esculetin ^b^	-	<LOQ	<LOQ	<LOQ	-	1.37	-	1.95
4	Caffeic acid ^a^	-	<LOQ	-	-	-	-	-	-
5	Ethyl caffeate ^b^	<LOQ	-	-	-	2.66	-	-	-
6	Caffeic acid prenyl ester (Prenyl caffeate) ^b^	-	5.01	-	-	-	2.47	-	1.72
7	Caffeic acid benzyl ester ^b^	-	<LOQ	-	-	-	1.43	-	-
8	Caffeic acid phenethyl ester (CAPE)^b^	-	6.28	-	-	-	<LOQ	-	8.35
9	Caffeic acid cinnamyl ester ^b^	-	1.98	-	-	-	<LOQ	-	12.03
10	Caffeic acid hexoside is. I ^b^	-	<LOQ	-	-	-	<LOQ	-	<LOQ
11	Caffeic acid hexoside is. II ^b^	-	2.17	<LOQ	-	-	<LOQ	-	<LOQ
Non-phenolic compounds									
12	Abscisic acid ^b^	3.06	7.98	13.03	<LOQ	1.85	6.36	-	-
∑ phenolic acid derivatives + abscisic acid		3.06	25.75	13.03	-	4.51	11.63	-	25.54
Phenolic acid amides (Phenylamides)									
13	Di-coumaroyl spermidine ^b^	-	-	-	5.41	-	-	-	7.41
14	Tri-coumaroyl spermidine ^b^	-	<LOQ	3.88	2.02	-	<LOQ	5.54	9.09
15	Dicoumaroyl caffeoyl spermidine ^b^	-	-	<LOQ	-	-	-	<LOQ	-
16	Tetra-coumaroyl spermidine ^b^	-	-	-	-	-	-	-	1.66
∑		-	-	3.88	7.43	-	-	5.54	18.16
Flavonoids and derivatives									
Flavonol aglycones and glycosides									
17	Galangin ^c^	80.27	119.72	9.10	15.34	23.47	47.68	-	107.30
18	Galangin-methyl-ether ^c^	5.59	5.20	<LOQ	-	-	-	-	-
19	Kaempferide ^c^	3.21	-	<LOQ	-	-	<LOQ	-	-
20	Quercetin ^a^	-	-	-	-	-	-	-	3.64
21	Isorhamnetin ^c^	-	6.38	9.85	-	-	3.95	9.09	6.97
22	Rhamnetin ^c^	-	4.21	-	-	-	1.19	-	1.14
23	Quercetin-dimethyl-ether is. I ^c^	-	2.33	-	-	-	1.21	-	6.69
24	Quercetin-dimethyl-ether is. II ^c^	21.37	25.02	8.59	-	-	-	-	-
25	Kaempferol-3-O-rhamnoside ^c^	-	4.56	-	-	-	-	-	-
26	Kaempferol-3-O-(6″-rhamnosyl)hexoside-7-O-rhamnoside ^c^	-	6.66	-	-	-	-	-	-
∑		110.45	174.06	27.54	15.34	23.47	54.03	9.09	125.74
Flavanonol aglycones and derivatives									
27	Pinobanksin ^c^	102.75	124.18	14.04	38.06	40.91	86.17	1.53	160.69
28	Pinobanksin-5-methyl-ether ^c^	16.89	53.33	5.68	3.63	9.96	27.76	-	86.76
29	Pinobanksin-3-O-propionate ^c^	-	-	-	-	-	-	-	5.15
30	Pinobanksin derivative ^c^	-	-	-	-	-	-	-	1.96
31	Pinobanksin-3-O-butyrate ^c^	-	-	-	-	-	-	-	18.40
32	Pinobanksin-3-O-pentanoate is. I ^c^	2.75	-	-	-	-	-	-	15.16
33	Pinobanksin-3-O-pentanoate is. II ^c^	15.40	3.36	-	-	-	-	-	11.99
34	Pinobanksin-3-O-hexanoate ^c^	-	-	-	-	-	-	-	5.34
∑		137.79	180.87	19.72	41.70	50.87	113.92	1.53	305.45
Flavone aglycones									
35	Chrysin ^a^	163.17	144.74	38.64	69.48	78.60	100.80	16.52	157.10
36	Apigenin ^a^	-	12.29	-	3.43	-	5.52	-	4.13
37	Genkwanin ^d^	-	9.84	-	-	-	4.87	-	30.67
38	Acacetin ^d^	50.67	37.43	5.00	13.65	17.28	22.47	-	75.98
39	Luteolin-methyl-ether ^d^	-	14.09	-	3.03	-	4.75	-	15.71
∑		213.84	218.38	43.64	89.59	95.88	138.41	16.52	283.59
Flvanone aglycones									
40	Pinocembrin ^a^	404.47	428.49	69.30	167.91	174.27	289.59	22.95	458.45
41	Pinostrobin ^e^	-	-	10.44	-	-	-	-	-
42	Sakuranetin ^e^	16.80	-	-	17.29	-	-	-	-
∑		421.27	428.49	79.74	185.20	174.27	289.59	22.95	458.44
∑∑		886.40	1027.57	187.55	339.25	349.00	607.58	55.62	1216.91

Abbreviations: Compound content expressed using available standards ^a^; Compounds expressed as caffeic acid equivalent ^b^; Compounds expressed as quercetin equivalent ^c^; Compounds expressed as chrysin equivalent ^d^; Compounds expressed as pinocembrin equivalent ^e^; <LOQ—less of limit of quantification; “-”—nonidentified/nonquantified phenolic compounds.

**Table 4:** Content of mineral elements of tested honey samples ^a^.

Element	Organic Produced				Conventionally Produced				Pooled Std
	Linden	Acacia	Chestnut	Meadow	Linden	Acacia	Chestnut	Meadow	
macroelements (μg/g)									
Ca	169.46 ^a^	15.70 ^b^	78.48 ^c^	25.49 ^d^	147.25 ^e^	15.29 ^f^	240.60 ^g^	121.36 ^h^	0.02
K	1248.81 ^a^	191.41 ^b^	1305.66 ^c^	1346.82 ^d^	1281.12 ^e^	183.91 ^f^	2225.56 ^g^	366.32 ^h^	2.04
Mg	23.73 ^a^	6.86 ^b^	52.31 ^c^	98.00 ^d^	22.56 ^a^	5.93 ^e^	51.39 ^c^	28.89 ^f^	0.54
Na	13.28 ^a^	12.99 ^a^	46.82 ^b^	13.13 ^a^	12.57 ^a^	12.84 ^a^	16.93 ^c^	14.28 ^d^	0.54
P	807.18 ^a^	895.24 ^b^	853.64 ^c^	923.92 ^d^	840.57 ^e^	795.40 ^f^	922.59 ^d^	874.92 ^g^	2.02
S	27.58 ^a^	15.65 ^b^	56.37 ^c^	95.36 ^d^	25.34 ^e^	17.61 ^f^	40.31 ^g^	33.07 ^h^	0.82
microelements (μg/g)									
Co	0.08 ^a^	n.d	n.d	0.02 ^b^	n.d	0.01 ^b^	n.d	n.d	0.008
Cr	0.05 ^a^	0.05 ^a^	0.06 ^a^	0.10 ^b^	0.06 ^a^	0.03 ^a^	0.10 ^b^	0.05 ^a^	0.02
Cu	0.11 ^a^	0.10 ^a^	0.41 ^b^	0.84 ^c^	0.09 ^ad^	0.07 ^d^	0.28 ^be^	0.18 ^e^	0.04
Fe	5.44 ^a^	1.10 ^b^	3.63 ^c^	3.59 ^c^	1.00 ^bd^	0.91 ^d^	1.52 ^e^	0.91 ^d^	0.09
Mn	2.22 ^a^	0.30 ^b^	6.60 ^c^	5.42 ^d^	0.67 ^e^	0.09 ^f^	3.48 ^g^	0.20 ^h^	0.05
Ni	0.05 ^ab^	0.08 ^b^	0.30 ^c^	0.47 ^d^	0.04 ^a^	0.08 ^ab^	0.12 ^e^	0.17 ^f^	0.04
Sr	0.59 ^a^	0.03 ^b^	0.19 ^c^	0.04 ^b^	0.11 ^d^	0.03 ^b^	0.22 ^c^	0.05 ^e^	0.03
Zn	1.19 ^a^	0.58 ^b^	0.74 ^c^	3.75 ^d^	0.38 ^e^	0.22 ^f^	1.23 ^a^	1.86 ^g^	0.04
toxic elements (μg/g)									
Al	1.39 ^a^	1.19 ^bc^	4.31 ^d^	2.75 ^e^	0.99 ^b^	1.16 ^c^	1.59 ^a^	1.21 ^bc^	0.05
As	n.d	n.d	n.d	n.d	n.d	n.d	n.d	n.d	-
B	2.24 ^a^	2.43 ^b^	1.71 ^c^	3.81 ^d^	2.90 ^e^	3.09 ^f^	2.27 ^a^	7.54 ^g^	0.04
Ba	0.27 ^a^	0.01 ^b^	0.23 ^a^	0.05 ^c^	0.05 ^c^	0.01 ^b^	1.11 ^d^	0.02 ^e^	0.02
Cd	n.d	n.d	n.d	0.01	n.d	n.d	n.d	n.d	0.002
Li	0.01 ^a^	0.009 ^a^	0.017 ^b^	0.008 ^a^	0.009 ^a^	0.007 ^c^	0.011 ^b^	0.010 ^b^	0.001
Pb	n.d	n.d	n.d	n.d	n.d	n.d	n.d	n.d	-
TMMEC (μg/g)	2299.77	1140.09	2405.21	2516.95	2331.76	1032.42	3504.33	1442.26	-
TTEC (μg/g)	3.91	3.64	6.27	6.63	3.95	4.27	4.98	7.57	-
TMEC (μg/g)	2303.68	2411.48	2411.477	2523.588	2335.71	1036.69	3509.31	1449.83	-

^a^ Means in the same row with different letters are a significant difference according to the *t*-test (*p* < 0.05). Data are expressed as mean and pooled standard deviation (Pooled std) of three replicates. n.d.-not detected. TMMEC- total macro- and micoelements content; TTEC—total toxic elements content; TMEC—total mineral content.

## Data Availability

The original contributions presented in the study are included in the article/Supplementary Material, further inquiries can be directed to the corresponding author.

## Supplementary Materials

The following supporting information can be downloaded at https://www.mdpi.com/article/10.3390/foods13223573/s1, Table S1. Botanical and geographical origin of examined honey samples; Table S2. Equation parameters of used phenolic standards for quantification; Table S3. Correlation coefficients (*r*) between quality parameters of tested honey samples; Table S4. Classification results of QDA model with 8 classes; Table S5. Classification results of QDA model with 4 classes (botanical origin); Figure S1. QDA discrimination plots; Figure S2. QDA discrimination plots.
