# Supplementary material for: Conventional vs. Organically Produced Honey—Are There Differences in Physicochemical, Nutritional and Sensory Characteristics?
Source: Foods. 2024 Nov 8;13(22):3573. doi: 10.3390/foods13223573 (PMC11593316; doi:10.3390/foods13223573)
Supplement: Supplementary file 1 [file foods-13-03573-s001.zip › foods-3266234-supplementary.pdf]

Supplementary material

Conventional vs. Organically Produced Honey—Are There Differences in Physicochemical, Nutritional and Sensory Characteristics?

Sladjana P. Stanojević [1,*], Danijel D. Milinčić [1], Nataša Smiljanić [1], Mirjana B. Pešić [1], Nebojša M. Nedić [2], Stefan Kolašinac [3], Biljana Dojčinović [4], Zora Dajić-Stevanović [3] and Aleksandar Ž. Kostić [1]

[1] Department of Food Technology and Biochemistry, Faculty of Agriculture, University of Belgrade, Nemanjina 6, 11080 Belgrade, Serbia
[2] Department of Breeding and Reproduction of Domestic and Farmed Animals, Faculty of Agriculture, University of Belgrade, Nemanjina 6, 11080 Belgrade, Serbia
[3] Department of Agrobotany, Faculty of Agriculture, University of Belgrade, Nemanjina 6, 11080 Belgrade, Serbia
[4] Institute of Chemistry, Technology and Metallurgy National Institute of the Republic of Serbia, University of Belgrade, Njegoševa 12, 11000 Belgrade, Serbia
* Correspondence: sladjas@agrif.bg.ac.rs

Table S1. Botanical and geographical origin of examined honey samples

beekeeping procedure	botanical origin	geographical origin	coordinates
	linden	Srbija	43°25′N 22°07′E
organic	acacia	Srbija	44°49′04″N 20°27′25″E
produced	chestnut	Greece	37°25'49"N 22°23'16"E
	meadow	Srbija	44°46'42.3"N 17°11.632' E
	linden	Srbija	45° 06' 60.00"N 19° 46' 59.99"E
conventionally	acacia	Srbija	44° 33' 59.99"N 20° 30' 59.99"E
produced	chestnut	Bosnia and Herzegovina	45° 02' 60.00"N 16° 01' 60.00"E
	meadow	Croatia	45° 46' 12.00"N 18° 36' 13.00"E

Table S2. Equation parameters of used phenolic standards for quantification.

Standards	Y=a*X±b	R^2	Linear range (ppm)	LOQ (ppm)
Caffeic acid	y= 3049527.6728 * x + 856403.3939	0.9921	0.1-10	3.65
Chrysin	y= 421642.8753 * x + 255584.8437	0.9914	0.1-10	3.88
Apigenin	y= 215501.5584 * x + 64892.2142	0.9942	0.1-8	2.93
Pinocembrin	y= 156751.2871 * x + 40416.1819	0.997	0.1-10	2.27
Quercetin	y= 769214.5670 * x + 825343.6827	0.9915	0.1-7	2.65

Table S3. Correlation coefficients (*r*) between quality parameters of tested honey samples[a]

parameters	TPC	TFC	DCA	CUPRAC	FRP	DPPH	pH	FA	M	colour	D	EC	OR
TPC	-												
TFC	0.63	-											
DHCA	0.57	0.82	-										
CUPRAC	0.41	0.25	0.21	-									
FRP	0.85	0.76	0.78	0.61	-								
DPPH	0.59	0.63	0.83	0.43	0.79	-							
pH	0.31	0.34	0.44	0.31	0.52	0.81	-						
FA	0.43	0.61	0.78	-0.18	0.53	0.34	-0.08	-					
M	-0.14	-0.43	-0.64	-0.43	-0.47	-0.38	0.08	-0.56	-				
colour	0.63	0.88	0.82	0.40	0.80	0.87	0.70	0.36	-0,28	-			
D	0.36	0.30	0.12	-0.42	0.11	0.14	0.27	0.16	0.63	0.35	-		

EC	-0.08	0.49	0.68	-0.22	0.34	0,37	0.21	0.78	-0.54	0.35	0.01	-	
OR	0.18	0.53	0.77	0.18	0.59	0.75	0.63	0.51	-0.57	0.59	0.15	0.81	-
TMC	0.40	0.39	0.46	0.09	0.55	0.73	0.93	0.14	0.19	0.66	0.48	0.35	0.63

[a]Significant et $p < 0.05$; TPC - total polyphenols content; TFC - total flavonoids content; DHCA - derivatives of hydroxycinnamic acid; CUPRAC - cupric reducing antioxidant capacity; FRP - ferric reducing power assay; DPPH$^{\cdot}$- DPPH radical scavenging activity assay; FA - free acidity; M - moisture content; D - diastasis; EC - electrical conductivity; OR - optical rotation; TMC - total mineral content.

Tables S4. Classification results of QDA model with 8 classes

		Predicted value							
	Groups*	1	2	3	4	5	6	7	8
Actual value	1	36	0	0	0	0	0	0	0
	2	0	36	0	0	0	0	0	0
	3	0	0	30	3	0	0	0	0
	4	0	0	6	33	0	0	0	0
	5	0	0	0	0	36	0	0	0
	6	0	0	0	0	0	36	0	0
	7	0	0	0	0	0	0	36	0
	8	0	0	0	0	0	0	0	36
Correct classified (%)		100	100	83.33	91.67	100	100	100	100
Overall (%)		96.88							

*Groups: 1-linden organic; 2-accacia organic; 3-chestnut organic; 4-meadow organic; 5-linden conventional; 6-accacia conventional; 7-chestnut conventionall; 8-meadow conventionall.

Table S5. Classification results of QDA model with 4 classes (botanical origin)

		Predicted value			
	Groups*	1	2	3	4
Actual value	1	64	1	0	1
	2	8	71	0	0
	3	0	0	70	9
	4	0	0	2	61
Correct classified (%)		88.89	98.61	97.22	84.72
Overall (%)		92.36			

*Groups: 1-linden; 2-accacia; 3-chestnut; 4-meadow.

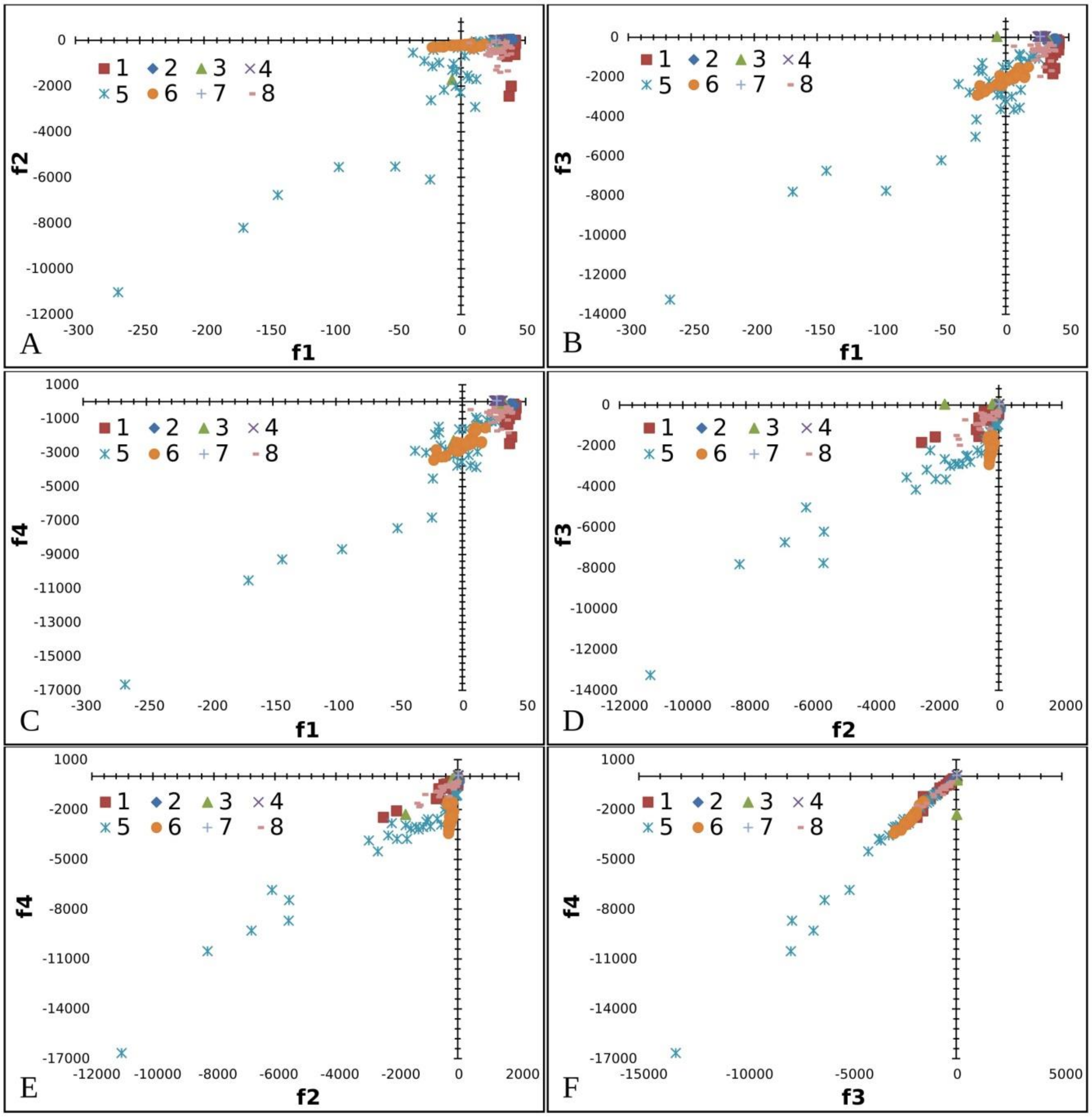
A
B
C
D
E
F
f1
f2
f3
f4
1 2 3 4
5 6 7 8

Figure S1. QDA discrimination plots: A, B, C, D, E and F ilustrate dicrimination between different honey samples. Discriminate scores (f1, f2, f3, f4) determine the sample's membership in an appropriate class. Legend: 1-linden organic; 2-accacia organic; 3-chestnut organic; 4-meadow organic; 5-linden conventionall; 6-accacia conventionall; 7-chestnut conventionall; 8-meadow conventionall.

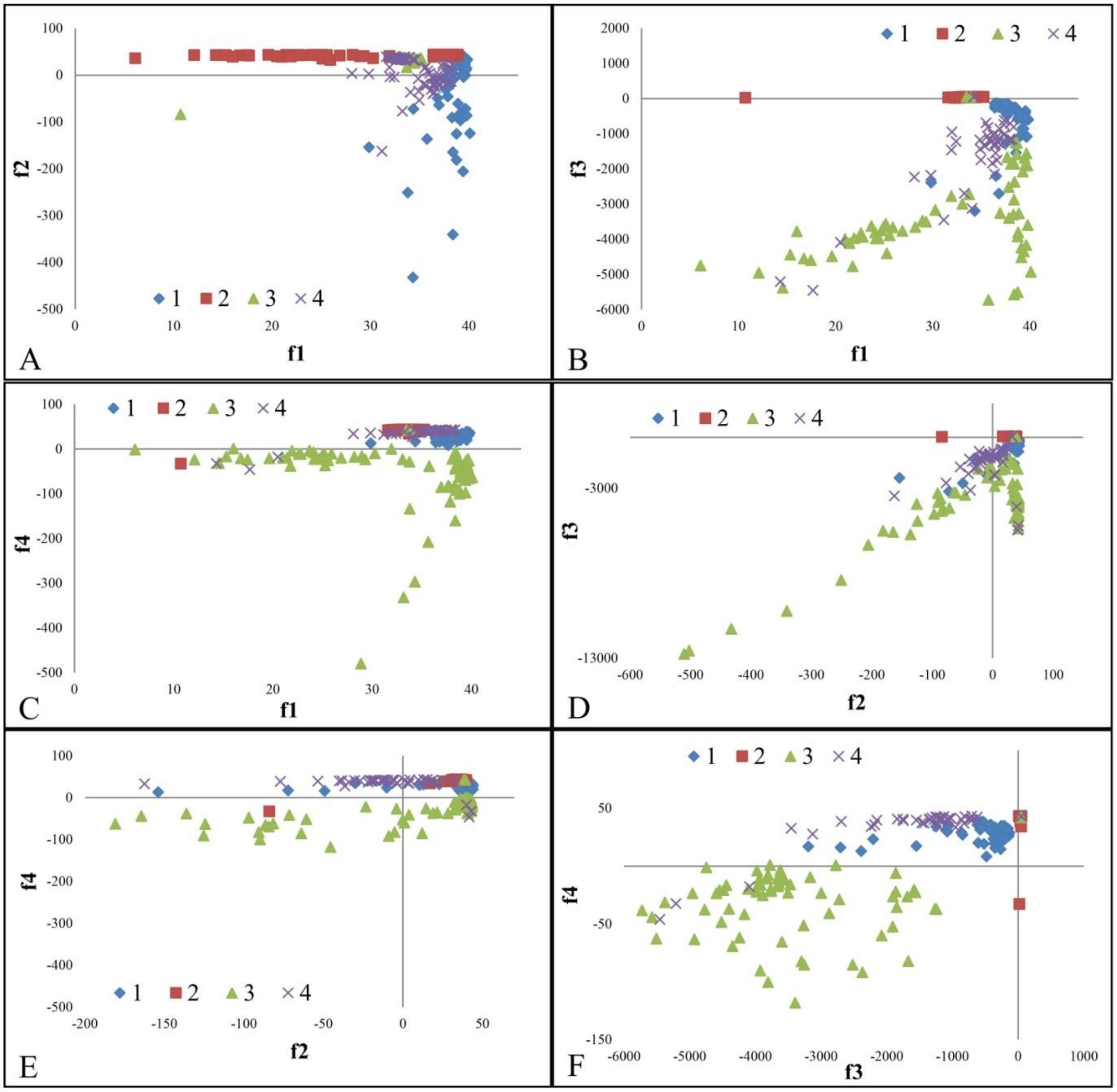

Figure S2. QDA discrimination plots: A, B, C, D, E and F ilustrate dicrimination between different honey samples. Discriminate scores (f1, f2, f3, f4) determine the sample's membership in an appropriate class. Legend: 1-linden; 2-accacia; 3-chestnut; 4-meadow.
